# Concurrent presence of buccal mucosal and ophthalmologic lesions in Behcet’s syndrome

**DOI:** 10.22088/cjim.9.4.325

**Published:** 2018

**Authors:** Mojtaba Mikaniki, Neda Babaee, Ebrahim Mikaniki, Mohammad Reza Hasanjani Roushan, Ali Bijani

**Affiliations:** 1 Student Research Committee, Babol University of Medical Sciences, Babol, Iran; 2 Dental Materials Research Center, Faculty of Dentistry, Babol University of Medical Sciences, Babol, Iran; 3 Social Determinants of Health Research Center, Health Research Institute, Babol University of Medical Sciences, Babol, Iran; 4 Infectious Diseases and Tropical Medicine Research Center, Health Research Institute, Babol University of Medical Sciences, Babol, Iran

**Keywords:** Behcet’s syndrome, Oral aphthous, Ocular lesion, Genital ulcers

## Abstract

**Background:**

Behcet’s syndrome is a disease with different aspects in its clinical manifestations. The purpose of this study was to evaluate the simultaneous presence of oral mucosal and ophthalmologic lesions in patients with Behcet’s syndrome.

**Methods:**

From April 2012 to December 2014, 50 cases of Behcet’s syndrome who referred to the Departments of Ophtalmology, Oral Diseases and Infectious Diseases of Babol University Medical Sciences were entered into the study. The diagnosis of the disease was performed using the Iranian criteria for the diagnosis of Behcet’s syndrome. The demographic findings as well as clinical manifestations were recorded.

**Results:**

Thirt-six (72%) were males and 14 (28%) were females. The mean age of the patients was 35.6±9 years. Oral aphthous (94%), ocular lesion (76%) and genital ulcers (70%) were the most clinical findings. The clinical onset of the disease in 43 (86%) was oral lesions and in 5 (10%) was ocular lesions. Among the forty-eight cases with oral lesions, 77% had ocular lesions concurrently. HLA-B5 was positive in 35 (70%) cases. Ocular lesion was seen in 33 of 35 (91.4%) cases versus 6 of 15 (40%) with HLA-B5 positive and negative cases, respectively (p<0.05). Oral lesion was seen in 94.3% cases with positive HLA-B5 and in 100% cases with negative HLA-B5 (p>0.05).

**Conclusions:**

The results show that concurrent ophthalmic and oral lesions in Behcet’s syndrome are relatively high. HLA-B5 positive cases are associated with more ophthalmologic lesions.

Behcet’s disease is a recurrent inflammatory disorder involving various organs of the body with unknown etiology and is characterized with triad of oral aphtose, genital ulcers and ocular involvement (1). In 1990, new diagnostic criteria including recurrence of oral lesions three times a year with two of the following findings were used for the diagnosis of the disease and these include: (uveitis or retinal vasculitis), recurrent genital ulcers, dermatologic findings like erythema nodosum, pseudofolliculitis and papulopustular lesions and positive pathergy test. The disease usually begins in the third decade of life and includes combinations of mucocutaneous, ocular, renal, cadiological, intestinal, pulmonary and neurological involvement (2). Other organs may be involved during the course of the disease as well. In Iran, the prevalence of the disease is 68 cases/100000 populations (3). Oral aphthous ulcer is painful with duration of 1-2 weeks. Ocular lesion is also associated with blindness and is the catastrophic finding during the course of the disease (4). The clinical manifestation of the disease is protean, so the purpose of this study was to evaluate the simultaneous presence of oral and ophthalmologic lesions in patients with Behcet’s syndrome in Babol, North of Iran.

## Methods

From April 2012 to December 2014, 50 cases of Behcet’s syndrome who referred to the Departments of Ophtalmology, Oral Diseases and Infectious Diseases were entered into the study. The diagnosis of the disease was performed using Iranian criteria for the diagnosis of Behcet’s syndrome (recurrent genital ulcers, ocular lesion including uveitis or retinal vasculitis, dermatological lesions including erythema nodosum, pseudofolliculitis and papular or pustular lesions and positive pathergy test). The presence of two of the above criteria was sufficed for the diagnosis of the disease. Demographic findings like age, sex as well as clinical manifestations in all cases were recorded. HLA-B5 also was assessed in all cases. Data were collected and analyzed. The difference for the development of ocular lesion with and without positive HLA-B5 cases was determined using X^2 ^test.

## Results

The mean age of these 50 patients (36 males, 14 females) was 35.6±9 years (ranged 13-55 years). The clinical manifestations of these patients are shown in table 1.

**Table 1:** The clinical manifestations of Behcet’s syndrome in 50 cases

**Clinical manifestation**	**No (%)**
Oral aphtous ulcer	48 (96)
Ocular lesion	38 (76)
Genital ulcer	35 (70)
Skin lesion	30 (60)
Cardiac involvement	5 (10)
Pulmonary involvement	9 (18)
Articular involvement	28 (56)

Renal, gastrointestinal and neurological involvement were seen in 6 (12%), 9 (18%) and 5 (10%) cases, respectively. The number of oral lesions were from 1 to 12 lesions. Among the patients with oral aphthous ulcers, 41 (82%) had painful lesion. The oral lesion was the initial clinical manifestation in 43 (86%) of cases. Ocular lesion was the initial manifestation in 5 (10%) of cases. Genital lesion was the initial clinical manifestation in 2 (4%) cases. HLA-B5 was positive in 35 (70%) cases. Ocular lesion was seen in 33 of 35 cases (91.4%) versus 6 of 15 (40%) with HLA-B5 negative (p<0.05). Oral lesion was seen in 94.3% cases with positive HLA-B5 and in 100% cases with negative HLA (p>0.05).

## Discussion

In this study, we assessed 50 consecutive patients with Behcet’s syndrome who were admitted to our centers. The sex distribution in our series was similar with the findings of other researchers (2, 5, 6). The mean age of our cases was higher than 26 cases reported by Omidian et al. and previously reported by Mikaniki et al. in 100 cases (3, 7). In the current study, the initial clinical manifestation was oral aphthous (86%) followed with ocular lesion (10%) and genital ulcer (4%) and was similar with the findings of other researchers (8, 9). 

The mean age at the onset of the disease was consistent with the findings of other researchers (2, 3, 6,10, 11). The number of oral lesions and its recurrence in our study was similar with the finding of Sanatkhany et al. and others (8, 9, 11, 12). Ocular lesions were found in 76% of our cases like the results of several reported studies (3, 13-16). But other studies reported ocular lesions in 30%-50% of their cases (7, 10, 17). Regarding genital ulcers, we found them in 70% of our series but other studies reported genital ulcers in 30%-84% of their cases (7, 9, 10, 18-21). The differences may be due to ethnicity, or other genetic factors. Regarding family history of the disease, we found it in 11(22%) of our cases but Shaker et al. and Al-Araji et al. reported 6.7% and 9.7% of their cases, respectively (22, 23). 

In this study, skin lesions were found in 60% of our cases. Our results were similar with the fundings obtained by other researchers (22, 24-26). An interesting finding in our study was more on the ocular involvement of those with HLA-B+ cases and a finding that was not reported previously. Further studies are required to confirm our results. In summary, the results show that a concurrent ophthalmic and oral lesion in Behcet’s syndrome is relatively high. HLA-B5 positive cases are associated with more ophthalmologic lesions.
